# Expression patterns and function of chromatin protein HMGB2 during mesenchymal stem cell differentiation

**DOI:** 10.1186/ar3669

**Published:** 2012-02-09

**Authors:** Noboru Taniguchi, Beatriz Caramés, Yasuhiko Kawakami, Martin Lotz

**Affiliations:** 1 Hakuaikai Kaisei Hospital, Obihiro, Hokkaido, Japan; 2 The Scripps Research Institute, La Jolla, CA, USA; 3 University of Minnesota, Minneapolis, MN, USA

## 

The superficial zone (SZ) of articular cartilage is critical in maintaining tissue function and homeostasis and represents the site of the earliest changes in osteoarthritis (OA). The expression of chromatin protein HMGB2 is restricted to the SZ, which contains cells expressing mesenchymal stem cell (MSC) markers [1]. Aging-related loss of HMGB2 and gene deletion are associated with reduced SZ cellularity and early onset OA [2]. This study addressed HMGB2 expression patterns in MSC and its role during differentiation.

**Figure 1:**
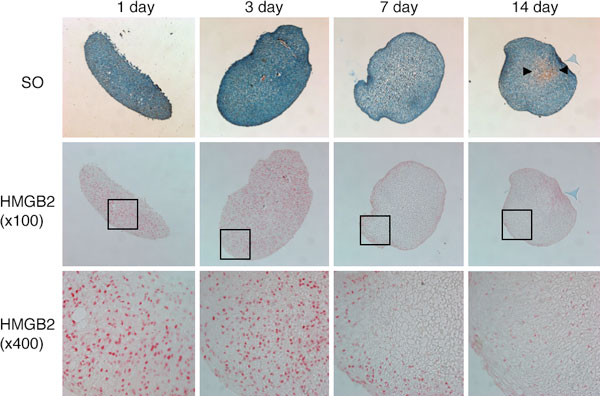
**HMGB2 expression during chondrogenesis of human MSC**. Immunohistochemistry shows that HMGB2 is expressed at days 1 and 3, but that expression is reduced at days 7, 14 upon induction of chondrogenesis. SO: safranin O staining.

HMGB2 was detected at higher levels in human MSC as compared to human articular chondrocytes and its expression declined during chondrogenic differentiation of MSC (Figure 1). Lentiviral HMGB2 transduction of MSC suppressed chondrogenesis as reflected by an inhibition of Col2a1 and Col10a1 expression. Conversely, in bone marrow MSC from Hmgb2-/- mice, Col10a1 was more strongly expressed than in wildtype MSC. This is consistent with in vivo results from mouse growth plates showing that Hmgb2 is expressed in proliferating and prehypertrophic zones but not in hypertrophic cartilage where Col10a1 is strongly expressed. Osteogenesis was also accelerated in Hmgb2-/- MSC. The expression of Runx2, which plays a major role in late stage chondrocyte differentiation, was enhanced in Hmgb2-/- MSC and HMGB2 negatively regulated the stimulatory effect of Wnt/β-catenin signaling on the Runx2 proximal promoter.

These results demonstrate that HMGB2 expression is inversely correlated with the differentiation status of MSC and that HMGB2 suppresses chondrogenic differentiation. The aging-related loss of HMGB2 in articular cartilage may represent a mechanism responsible for the decline in adult cartilage stem cell populations.
